# Contemporary Theory and Research

**Published:** 1893-04-15

**Authors:** 


### Contemporary Theory and Research.

### TOO MANY DOCTORS.

The *Riforma Medica* complains that the number of doctors in Italy is already in excess of the number required, and is continually increasing. Among other large towns Naples counts a doctor for every 513 inhabitants. The natural result is that the amount of fees which falls to the lot of these practitioners is very inconsiderable. Compared with all the other liberal professions it would seem that medicine is, from a pecuniary point of view, the least productive in Italy.

### RURAL MEDICAL AID IN FRANCE.

M. Theophile Roussel has been calling attention to the necessity for some organised system of medical relief for the country population in France. According to the report most of the peasants are at the present moment abandoned to their sad lot without care and without protection. Medical aid is only established in the departments and communes sufficiently rich to meet the necessary expense. One of the causes of the constant depopulation of the country districts in favour of the towns appears to be the absence of any kind of public assistance in the villages. A remarkable report has also been issued with respect to hospital administration in country districts, winding up with the surely not unjustifiable demand that the under officials of the medical service in these hospital establishments shall not be engaged until they have given evidence of possessing sufficient knowledge to undertake the care of the sick and wounded. M. Monod, Director of the Assistance Publique, admitting all the criticisms to be just, complains that his administration is powerless to produce the slightest improvement in these hospitals, since each time an unfavourable report on the state of one of them reaches the Direction it is transmitted to the Administrative Commission on which this depends, and the matter ends with the reply that it is impossible to make any change for want of funds.

### NEW ENEMIES OF THE NERVES.

Dr. Monin has observed that telegraphists and telephonists exposed to frequent and repeated magnetic irradiations are specially subject to nervous spasms, to vertigo, and cerebral disorders. In complaining of the irregularity of telephone officials, it must be remembered that it is a truly "internal" profession. However little nervous a man may be, at the end of rather frequent telephonic auditions, he experiences irritation, giddiness, exhaustion, and painful humming in the ears. Judge after that of the degree of fatigue and nervous exhaustion which must be experienced by a young girl whose attention and hearing are thus on the strain for twelve hours a day and thirty days a month. Dr. Gellé has also quoted several cases of extreme impressionability, with neuralgic pains and gradual loss of the injured sense of hearing in these Parisian telephone clerks. The effect of dynamite on the nerves requires little explanation, but is nevertheless remarkable. It has been long noticed that after the explosion of dynamite in water the fish nearest to the cartridge were stupified, while those at a greater distance turned over on their backs and floated on the surface of the water. They were not dead, however, for as soon as they were touched they darted off quickly; only they were in such a complete state of shock as to be deprived of all sense. The same effect is observable in the air after an explosion of dynamite, which is set in motion by such violent vibrations, that transmitted to the nervous centres they stun a man if he is sufficiently near or agitate him so violently that he is half-stupified. The unhappy Véry Hamonot and those who were in the restaurant at the moment of the explosion offer striking examples, and it is not doubtful that the nervous shock inflicted on their system had much to do with the unhappy result of the contusions or operations which they underwent.

